# Assessing New York City’s COVID-19 Vaccine Rollout Strategy: A Case for Risk-Informed Distribution

**DOI:** 10.1007/s11524-024-00853-z

**Published:** 2024-04-05

**Authors:** Nina Schwalbe, Marta C. Nunes, Clare Cutland, Brian Wahl, Daniel Reidpath

**Affiliations:** 1 School of Pathology, Faculty of Health Science, University of the Witwatersrand, January 1 Smuts Avenue, Braamfontein, Johannesburg, 2000 South Africa; 2 Heilbrunn Department of Population and Family Health, Columbia University, 722 W 168Th St, New York, NY 10032 USA; 3 Medical Research Council, Vaccines & Infectious Diseases Analytics Research Unit, Faculty of Health Sciences, University of the Witwatersrand, Johannesburg, 2000 South Africa; 4 Center of Excellence in Respiratory Pathogens (CERP), Hospices Civils de Lyon (HCL), and Centre International de Recherche en Infectiologie (CIRI), Team Public Health, Epidemiology and Evolutionary Ecology of Infectious Diseases, Université Claude Bernard Lyon 1, Inserm U1111, CNRS UMR5308, ENS de Lyon, Lyon, France; 5 Wits African Leadership in Vaccinology Expertise (Wits-Alive), School of Pathology, Faculty of Health Science, University of the Witwatersrand, Johannesburg, South Africa; 6 Department of International Health, Johns Hopkins Bloomberg School of Public Health, 615 North Wolfe Street, Baltimore, MD 21205 USA; 7 Institute for Global Health and Development, Queen Margaret University, Edinburgh, EH21 6UU UK; 8 School of Social Sciences, Monash University, Clayton, VIC 3125 Australia

**Keywords:** Vaccination, Vaccines, COVID-19, Immunization, New York City, New York, Risk factors, Health policy, Social risk factors, Spacial analysis, Urban health, Aged, SARS-CoV-2, Economic status, Poverty, Vaccination coverage, Morbidity, Coverage, COVID-19 vaccines, Censuses

## Abstract

This study reviews the impact of eligibility policies in the early rollout of the COVID-19 vaccine on coverage and probable outcomes, with a focus on New York City. We conducted a retrospective ecological study assessing age  65+, area-level income, vaccination coverage, and COVID-19 mortality rates, using linked Census Bureau data and New York City Health administrative data aggregated at the level of modified zip code tabulation areas (MODZCTA). The population for this study was all individuals in 177 MODZCTA in New York City. Population data were obtained from Census Bureau and New York City Health administrative data. The total mortality rate was examined through an ordinary least squares (OLS) regression model, using area-level wealth, the proportion of the population aged 65 and above, and the vaccination rate among this age group as predictors. Low-income areas with high proportions of older people demonstrated lower coverage rates (mean vaccination rate 52.8%; maximum coverage 67.9%) than wealthier areas (mean vaccination rate 74.6%; maximum coverage 99% in the wealthiest quintile) in the first 3 months of vaccine rollout and higher mortality over the year. Despite vaccine shortages, many younger people accessed vaccines ahead of schedule, particularly in high-income areas (mean coverage rate 60% among those 45–64 years in the wealthiest quintile). A vaccine program that prioritized those at greatest risk of COVID-19-associated morbidity and mortality would have prevented more deaths than the strategy that was implemented. When rolling out a new vaccine, policymakers must account for local contexts and conditions of high-risk population groups. If New York had focused limited vaccine supply on low-income areas with high proportions of residents 65 or older, overall mortality might have been lower.

## Introduction

On December 11, 2020, the USA granted emergency authorization for newly developed COVID-19 vaccines to prevent severe morbidity and mortality [1]. Three days later, on December 14, New York administered its first vaccines to high-risk hospital workers. This was followed in the state, in order, by nursing home workers and residents (December 21, 2020), all hospital workers (January 4, 2021), essential workers, and all adults 75 years and older (January 11, 2021), all adults (18 years and older) with underlying health conditions, including obesity (February 15, 2021), all adults 60 years and older (March 10, 2021), public-facing government and non-profit workers (March 17, 2021), all adults 50 years and older (March 23, 2021), all adults 30 years and older (March 30 2021), and then all adults 16 years and older (April 6, 2021) [2].

During this period, the New York State Department of Health (NYSDOH), in collaboration with the New York City Department of Health and Mental Hygiene (NYCDOH), delivered vaccines through fixed-point mass vaccination sites. Appointments were arranged through an English-language internet-based system [3], based on self-verification that applicants met age, health, or employment-related criteria.

The rapid pace with which vaccine eligibility was widened during the early months of 2021 may have been a rational choice had there been adequate availability of vaccines. However, limited stocks meant groups at lower risk were added to the pool of eligible recipients before those at higher risk were vaccinated [4, 5]. There were many reports about difficulties faced by people 65 years and older getting appointments to receive even their first dose [6–9].

In New York, as elsewhere, the probability of dying from COVID-19 was not equally distributed across the population. The single greatest risk factor for COVID-19-related mortality was older age [10–12]. Low-income households were also particularly vulnerable [13].

In light of these two equity-related vulnerabilities, this analysis explores whether New York widened vaccination eligibility too quickly in the face of vaccine shortages rather than focusing first on those at higher risk [14, 15]. The research aimed to answer the question of whether older New Yorkers living in low-income communities would have been better served in the face of COVID-19 vaccine shortages if distribution had been targeted toward them.

We reviewed differences in coverage of people 65 years and older (65 +) between the highest and lowest wealth quantiles just before the state expanded eligibility to younger age groups but when vaccines were still supply-constrained. We then examined whether differences persisted when vaccines were no longer supply-constrained. We also analyzed the relationship between risk factors, vaccination coverage, and COVID-19 mortality rates by geography.

## Methods

### Type of Study

We conducted a retrospective analysis of linked Census Bureau data and New York City Health administrative data aggregated at the level of modified zip code tabulation areas (MODZCTA). All analyses were done with R software (version 4.1.3). The authors complied with STROBE guidelines [16].

### Data Collection

Data on vaccination coverage, stratified by age and MODZCTA, were obtained by special request from the NYCDOH [17] through the Open Data initiative [18]. Race, income, and age data by MODZCTA were obtained from the US Census Bureau [19]. New York City (NYC) is divided into 177 MODZCTAs [20]. While a MODZCTA comprises either a single Zip Code Tabulation Area (ZCTA) or a combination of contiguous ZCTAs, no ZCTA contributes to more than one MODZCTA.

#### Demographic Data

Population data, disaggregated by age and sex, were drawn from the 2020 American Community Survey (ACS) 5-Year Estimates Detailed Tables [21]. The data available for each ZCTA were aggregated up to MODZCTAs using the mapping described and grouped into five age groups: 0–17, 18–24, 25–44, 45–64, and 65 + , to match age-grouped vaccination NYCDOH data. 23 ZCTAs had populations of 0 and were therefore excluded [22].

#### Wealth Data

Median household wealth data for each NYC ZCTA were drawn from the 2020 ACS 5-Year Estimates Detailed Tables and aggregated up to MODZCTAs. The three ZCTAs with very small populations (*n* = 44, 96, 220) for which median household income was unavailable, were treated as having zero median household income. Median income was aggregated up to MODZCTA as a population-weighted sum of the median income of each contributing ZCTA.

#### Vaccination Coverage Data

NYCDOH provided data on the percentage of the population in each MODZCTA that received at least one COVID-19 vaccine dose stratified by selected age groups and date. We focused on vaccination coverage for the week of March 27, 2021; before this, people less than 50 were not generally eligible for vaccination.

#### Mortality Data

NYCDOH provided the total number of COVID-19 deaths in each MODZCTA from December 1, 2020, to December 31, 2021. These data were not stratified by age. To preserve patient privacy, NYCDOH did not provide the number of deaths in any MODZCTA with less than ten deaths. As disaggregation by any factor (e.g., age, sex, gender) would increase the cells with mortality counts less than ten, and thus missing data, we opted for unstratified total mortality. Eleven (6.2%) of all 177 MODZCTAs recorded less than ten deaths. The minimum number of deaths in a MODZCTA was zero; the maximum was 302. We replaced missing data with a mid-point value of five because zero was definitively zero and any number ≥ 10 was definitively that number. Thus, missing data were less than 10 but more than zero. While we considered estimating the number of deaths by calculating the city death rate and multiplying that by the population of the MODZCTA (i.e., mean replacement), that would have resulted in 6 of the 11 missing counts exceeding 10, which was not possible. An examination of the distribution of death counts in the city’s MODZCTA’s also showed wide variation suggesting that a single MODZCTA that was within 5-points of its true value would adequately represent the true value within the model. Choosing other values did not noticeably change the models.

Combining mortality and population data, we calculated COVID-19 mortality rates per 100,000 population in each MODZCTA from December 1, 2020, to December 31, 2021.

### Data Analysis

#### Vaccination Coverage

We first explored vaccine coverage by MODZCTA wealth quintiles. Using age-group specific population and the vaccination coverage by MODZCTA, we calculated the number of doses distributed to each age group to assess, given supply constraints, the extent of “misallocation” (i.e., the excess doses, had vaccine supply been allocated exclusively to older individuals).

#### Mortality

We conducted an ecological analysis looking at the relationship between MODZCTA-specific mortality from December 1, 2020, to December 31, 2021, and the percentage of the population 65 + , the vaccination coverage in the 65 + , and the median household wealth. These three variables were centered to enhance stability of the models.

We estimated the relationship using OLS regression. We used Moran’s I to estimate spatial dependency in the residuals, which was significant. We used a spatial error model to estimate the bivariate relationships between (1) area-level proportion of individuals 65 + and COVID-19 mortality, (2) area-level median household income and COVID-19 mortality, and (3) area-level vaccination coverage 65 + and COVID-19 mortality [23]. In the full model, we started with a “full factorial” approach, including all the individual predictors and their interactions. We then looked at the effect of removing higher-order interactions, using an ANOVA test, and found removing any interaction effect resulted in a model with a significantly poorer fit. Based on an analysis of the residuals in the first step, we also tested the inclusion of a dummy for one outlier MODZCTA.

### Ethical Considerations

This study received ethical approval from the University of the Witwatersrand’s Human Research Ethics Committee (HREC), which determined that the research did not involve human subjects as defined by the university’s policies and federal regulations. Therefore, informed consent was not required for participation. The data were completely anonymized, and NYCDOH did not provide the number of deaths in any MODZCTA with less than ten deaths.

## Results

### Descriptive

Table 1 shows the variation across NYC MODZCTAs of the total population, proportion 65 + , median household income, mortality rate, and vaccination coverage by wealth quintile on March 27, 2021, and March 19, 2022.

**Table 1:** Summary statistics and vaccination coverage by March 19, 2022 (by MODZCTA)

Summary statistics	Minimum	Median	Mean	Maximum	IQR
Total population	3260	43,173	47,693	108,661	27,672–67,989
Proportion population 65 +	< 1	14.5	15.1	31	11.7–18.3
Median household income	$23,337	$74,042	$81,381	$250,001	$56,908–$96,787
COVID-19 mortality rate per 100,000 (2021)	0	126.8	127.1	397	93.2–151.3
Vaccination coverage at week March 27, 2021					
Lowest wealth quintile 65+	36.1	53.7	52.8	67.9	47.4–57.8
Highest wealth quintile 65+	41.2	73.6	74.6	99.0	64.0–82.8
Vaccination coverage at week March 19, 2022					
Lowest wealth quintile 65+	65.6	87.4	86.8	99	79–100
Highest wealth quintile 65+	52.1	94.6	90.2	99	82.5–99.0

The difference in the total population between the smallest and largest MODZCTAs ranged from 3260 to over 108,000 people. There was a 30-fold range in proportion of 65 + (less than 1 to 31%) and a tenfold range in median household income ($23,337 to $250,001). COVID-19 mortality in 2021 was four times higher in the highest versus lowest mortality area (397/100,000 vs 94/100,000).

### Vaccination Coverage

We explored the relationship between age-wealth, age-vaccination coverage, and wealth-vaccination coverage across MODZCTAs, focusing on vaccination coverage by the last week of March, just before everyone aged 30 + became eligible for vaccination.

The mean vaccination rate for 65 + ranged from 52.8% in the poorest quintile to 74.6% in the wealthiest. The maximum coverage was 99.0% among those 65 + in the wealthiest quintile versus 67.9% in the poorest. For those 45–64 years, the difference in mean coverage between the wealthiest and poorest quintiles was 25% (60% versus 34.6%). A year later, when vaccines were widely available, 65 + residents had median vaccination coverage exceeding 87%, including in the lowest wealth quintile (Table 1).

**Fig. 1:**
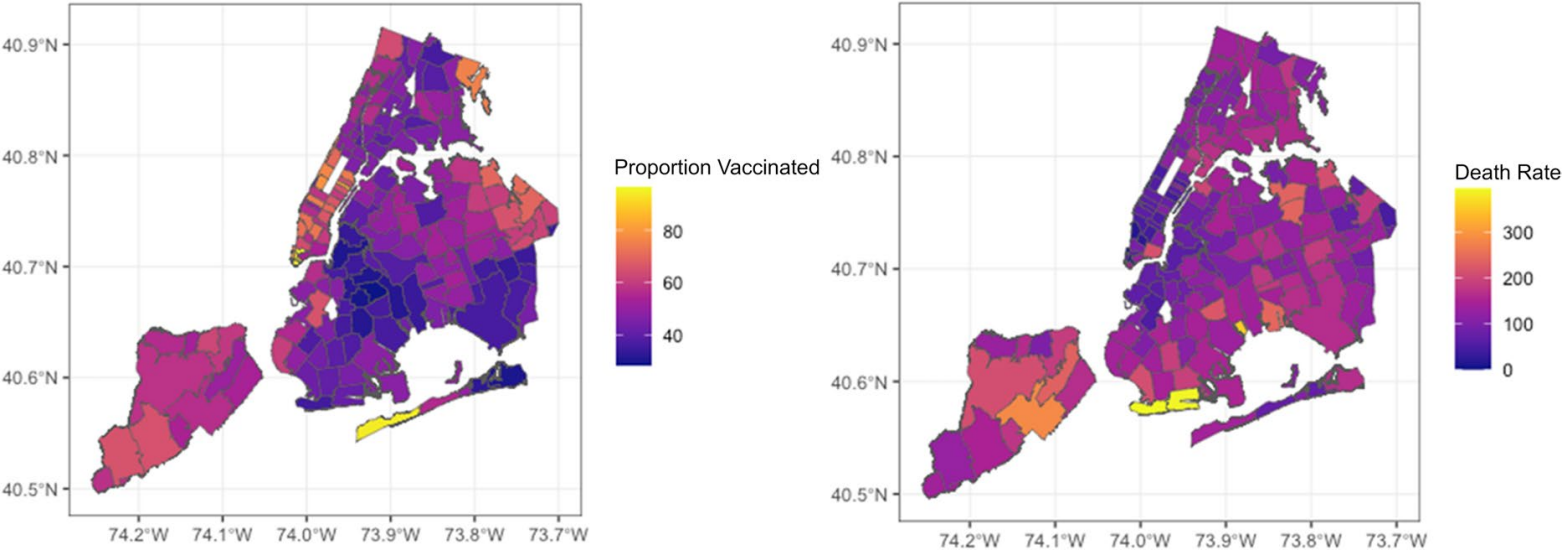
Proportion vaccinated with at least one dose by MODZCTA  65+ by the week of March 27, 2021 (left) and overall death rates by MODZCTA 2021 (right)

The proportion of 65 + vaccinated three months following vaccine introduction and the overall death rates by geographical area for 2021 are almost mirror images with opposite polarity. Areas with a higher proportion of 65 + vaccinated tended to have lower death rates, suggesting an inverse spatial relationship between the proportion vaccinated and the death rate (Fig. 1).

**Fig. 2:**
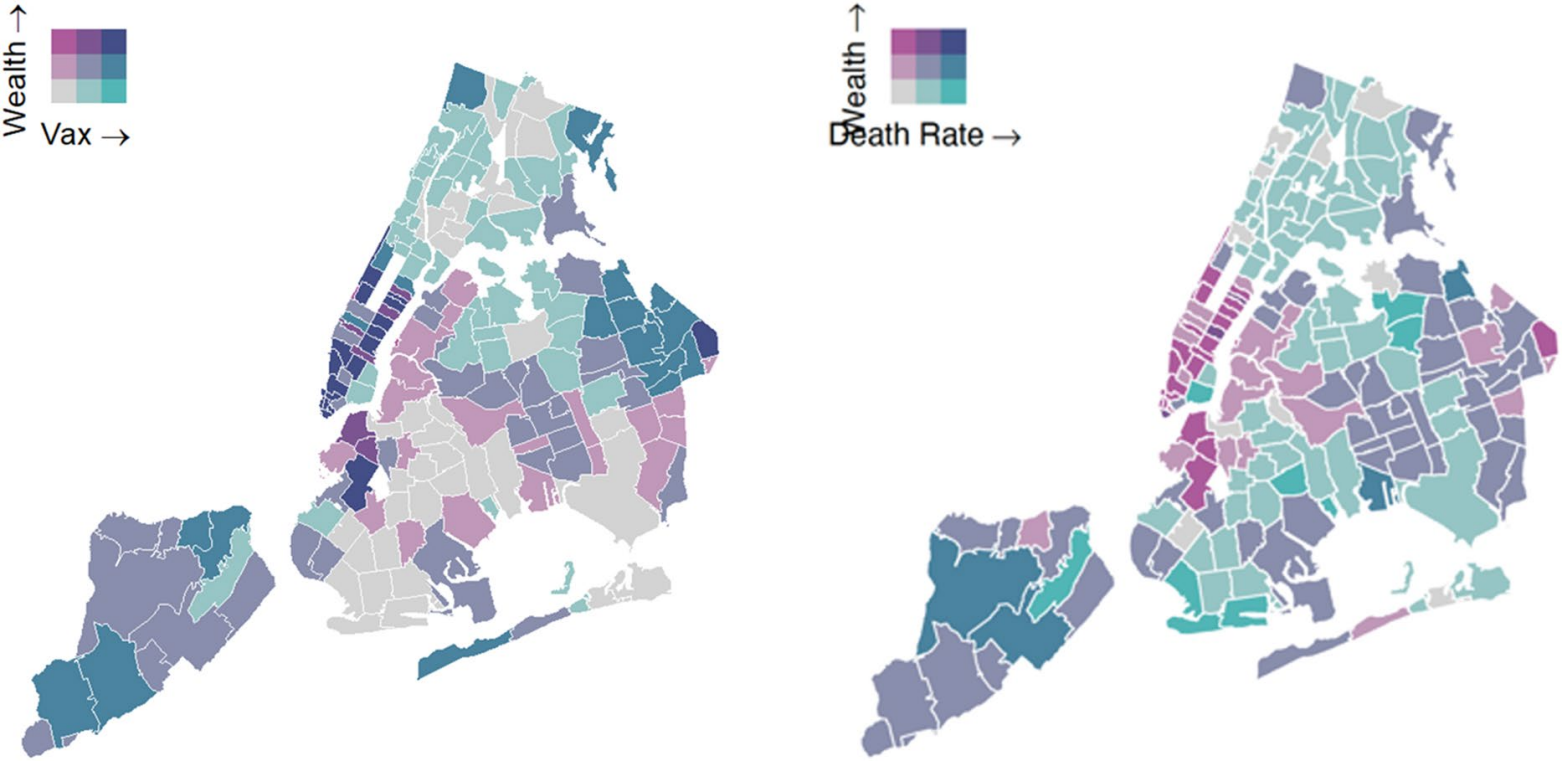
Wealth and  65+ vaccination rate (left) and wealth and mortality rate (right)

We also observed a positive correlation between weighted median income (wealth) and vaccination coverage for 65 + and an inverse correlation between wealth and mortality (Fig. 2).

**Fig. 3:**
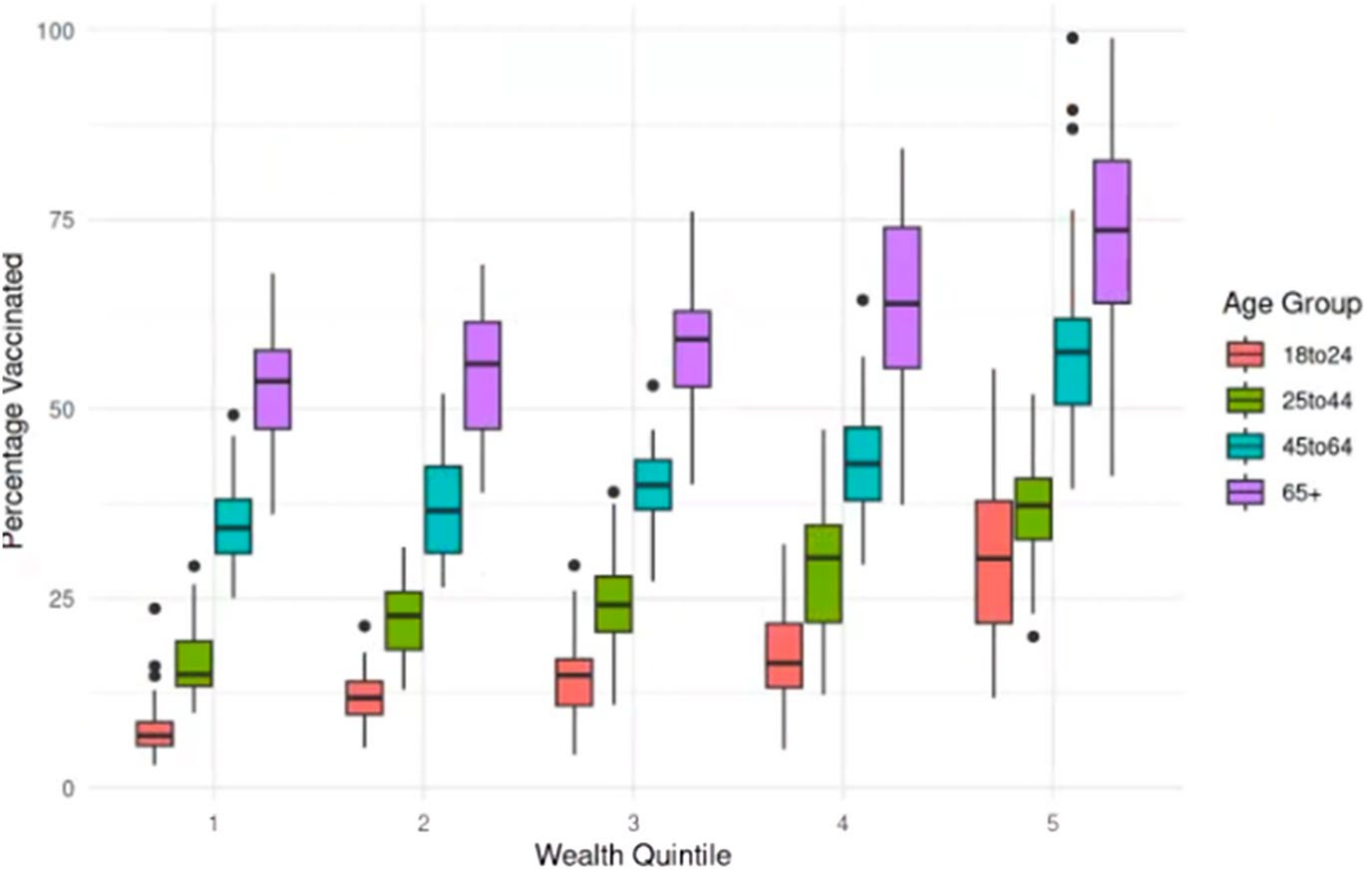
Percentage vaccinated by wealth quintile and age group

Figure 3 shows a boxplot of vaccination coverage, by age group, for each income quintile by March 27, 2021. The plot shows a consistent pattern whereby, for any age group, there is a monotonic and increasing relationship between wealth and vaccination coverage. However, as up to March 27, 2021, the vaccine was not generally available to people under 50; it is striking that (i) so many younger people were being vaccinated and (ii) wealthier people had higher coverage across all age groups.

We reanalyzed vaccination coverage using absolute numbers of doses to assess the potential “misallocation” of vaccines from older to younger age groups. We used coverage rates to derive doses delivered by age group by multiplying coverage rates by the population, per MODZCTA. This provided the minimum number of doses available during this period. From this, we identified that by March 27, 2021, MODZCTAs in the wealthiest quintiles had received enough vaccines to cover 41.6% of their total population. Vaccine availability dropped monotonically by wealth quintile: 31.6% coverage for the second wealthiest, 27.3% coverage for the middle, 24.9% for the second poorest, and 20.7% coverage for the poorest quintile.

We estimated the “misallocated doses” as any doses received by a person under 45 years before March 27, 2021, as by this date, only people older than 50 years were eligible for vaccination unless they were in high-risk health or employment categories. As described in the discussion section, we would expect to observe a larger proportion of these individuals living in the poorest quintiles and commensurate “overallocation.”

The greatest estimated overallocation of doses, however, occurred in the wealthiest quintiles.

Among them, 38.6% of doses were “misallocated” from older (65 +) to younger (0–17, 18–24, 25–44) age groups. The “misallocation rate” dropped monotonically by wealth quintile.

Moreover, if vaccination had been restricted to 65 + , there would have been enough vaccines for this age group in NYC 1.19 times over. By quintile, if in the poorest all doses delivered had only been administered to older New Yorkers, there would only have been doses to vaccinate 96.4% of the 65 + age group. In contrast, in the wealthiest, if vaccinating only older adults, there would have been 69% excess doses.

### Mortality

To explore the statistical relationship between mortality rates and the centered values for the percentage of the population 65 + in each MODZCTA, the vaccination coverage in the 65 + , and the median household income, we fit a “full factorial” OLS regression model. This model enables the study of the effects of multiple factors simultaneously on a response variable.

The Moran *I* test of the residuals, based on spatial autocorrelation, revealed strong evidence of positive spatial autocorrelation (*I* = 0.299, *p* < 0.0001), implying that locations close together exhibited values more similar than expected by chance alone.

To overcome the spatial correlation in the error observed in the initial model, we used a spatial error model. We tested the residuals using a Monte Carlo version of the Moran *I* test, which detected no significant spatial autocorrelation (*I* =  − 0.053, *p* = 0.799). The absence of significant spatial autocorrelation in the residuals suggests that the spatial error model successfully accounts for the spatial dependence present in the data. The model results, including coefficient estimates and statistical significance, are shown in Table 2.

**Table 2:** Spatial error regression with centered predictors

Predictor	Estimate	Std. error	*z* value	*P* value
Intercept	125.23	4.56	27.4573	< 0.0001
%Age 65+	5.8227	0.6996	8.3224	< 0.0001
Median income	− 1.0944	0.1144	− 9.5647	< 0.0001
%Vaccinated (65+)	− 0.3056	0.3241	− 0.9428	0.3458
Interaction terms	146.38	30.618	4.7809	< 0.0001
%65+ × median income	− 0.0971	0.0157	− 6.1686	< 0.0001
%65+ × %vaccinated	− 0.0239	0.0506	− 0.4725	0.6366
Income × %vaccinated	0.0233	0.0060	3.8723	0.0001
%65+ × income × %vaccinated	0.0025	0.0008	3.0352	0.0024

Except for one MODZCTA (with an exceptionally higher-than-average proportion of older adults) [24] mortality was higher in lower-income areas and areas with a greater proportion aged 65 + . Those areas were also less vaccinated.

The fitted spatial error model provides insights into the factors affecting the COVID-related mortality rate in MODZCTAs. First, assuming a MODZCTA with centered values for income ($74 K), percentage 65 + (14.5%), and vaccination rate in 65 + (14.5%), the expected mortality rate for the period 2021 is 125.2 per 100,000 people.

The coefficient for the percentage of the population 65 + was positive and statistically significant (*b* = 5.82, *p* < 0.001), indicating that, with centering, a 1% increase in the percentage of 65 + is associated with a 5.8% increase in the mortality rate (*p* < 0.0001), holding all other variables constant. Median household income had a negative and significant effect (*b* =  − 1.09, *p* < 0.001); a $1000 increase in median income is associated with a 1.1% decrease in the mortality rate (*p* < 0.0001), holding all other variables constant. A 1% increase in vaccination coverage in the 65 + age group is associated with a small (*− *0.3056; *p* = 0.3458) decrease in the mortality rate. While not statistically significant, it is in the expected direction.

The vaccination coverage among 65 + had a negative but non-significant coefficient (*b* =  − 0.31, *p* = 0.35), aligned with the protective effect of vaccination against mortality risk.

There were significant interactions between the percentage of 65 + , median income, and vaccination coverage. The negative interaction between the percentage of 65 + and income (*b* =  − 0.10, *p* < 0.001) indicated that the mortality effect of a larger elderly population diminished in higher-income areas. The positive income by vaccination coverage interaction (*b* = 0.02, *p* = 0.001) suggested that vaccination played a greater role in lowering mortality in higher-income regions. Finally, the three-way interaction between the percentage of 65 + , income, and vaccination coverage was positive and significant (*b* = 0.003, *p* = 0.002), implying a complex relationship between age, income, vaccination, and mortality.

While coefficient estimates were robust, accounting for spatial autocorrelation in the error term improved model fit and controlled for potential bias. Formal interpretation of the spatial structure is necessarily limited.

## Discussion

Income and age were clear predictors of mortality due to COVID-19 in the USA [14, 25, 26], where over 81% of COVID-19 deaths occurred in people aged 65 + [27]. Delaying vaccination in these groups resulted in avoidable deaths [28]. We used high-quality, public sector data to appraise the rollout of COVID-19 vaccination and COVID-19 mortality in NYC, a densely populated urban center with a widely ranging socio-economic and demographic contexts. We timed our analysis to maximize the ability to appraise vaccine uptake when vaccines were theoretically restricted to high-risk groups.

We found that low-income areas with high proportions of people 65 + demonstrated lower coverage rates (mean vaccination rate 52.8%; maximum coverage 67.9%) than wealthier areas (mean 74.6%; maximum 99%, in the wealthiest quintile) in the first 3 months of vaccine rollout, and higher mortality over the year following its introduction. These findings are consistent with the literature on the effects of age and wealth on COVID-19 mortality [13, 14, 29].

At a time when vaccine supply was still limited [30], many lower-risk, younger people accessed vaccines ahead of schedule, particularly in high-income areas (mean coverage 60% among those 45–64 years in the wealthiest quintile), suggesting “misallocation” of doses that could have been provided to older people, who had higher case fatality rates [31]. This could have been corrected by authorities through more stringent enforcement of state-mandated guidelines on distribution criteria.

While there is some plausibility that access for younger people was granted to those in scheduled professions or having underlying health risks, this is unlikely to account for the magnitude of difference between low and high-income MODZCTAs. Moreover, few working in such professions would fall into the wealthiest quintile. As poorer people make up the largest numbers of essential workers and are more likely to have underlying risk conditions and thus be eligible for the vaccine, we would assume vaccination rates in younger adults should have been higher in poorer areas [14]. This, however, was not the case suggesting that many people jumped the queue, with no consequences from the state.

Of note, when vaccines were no longer in short supply, uptake for all income groups aged 65 + rose above 80%. This suggests that vaccine access drove low coverage in the early months of the vaccine rollout.

Our findings demonstrate the direct results of failure to prioritize health resources according to risk in a public health emergency and underscores the importance of shepherding doses and prioritizing known high-risk groups in future. Across the US, income data are available by zip code, and Medicare and Medicaid have detailed records of household age and related medical conditions. Information on household age and composition is also available from voter registration data. These and other available data enable to target at-risk populations first and deploy a “precision public health” approach [32].

### Limitations

This study had several limitations. First, the mortality model is ecological and does not permit inferences about the association at the individual level. This limitation is further complicated by the fact that the mortality rate was based on the cumulative COVID-19 mortality for all ages in 2021, though the group of interest was 65 + . For data analytic purposes, we replaced the missing deaths with a mid-point value of five. Sensitivity analyses suggested that this had little effect on the final analysis.

The mortality data provided by NYCDOH was also for December 1, 2020, to December 31, 2021, including 1 month of deaths before vaccine availability. However, after an initial peak in April 2020, the number of deaths only started to increase again rapidly in January 2021, with a steady rise throughout that year.

Also, while we explored the relationship between vaccination coverage in the week of March 27, 2021, and COVID-19-associated mortality rates between December 2020 and December 2021, many aspects of COVID-19 were dynamic (e.g., the emergence of variants of concern, prevalence of infection-induced immunity) and could have affected mortality.

Due to data availability, we used area-level measures of vaccination coverage for the 65 + for March 2021 and the percentage of 65 + in MODZCTA populations. The challenge of interpreting the higher interaction terms of the mortality model may be attributable to this and could be overcome with the availability of better mortality data. Finally, we note that sex-disaggregated vaccination coverage data by geography was not available thus, gender is not considered in this analysis. This is an important limitation in assessing vaccine coverage and outcomes.

We note that lack of data and the ecological nature of the study meant that we could not explore a range of potential confounders including lack of legal documentation [33], lack of primary health care provider [34], languages spoken, and access to transport [35]. In NYC, these are all highly correlated with income.

We were also unable to explore potential factors contributing to hesitancy within New York City and between groups (e.g., race, ethnicity, religion). It is plausible, for example, that vaccine-hesitant people could have taken a “wait and see” approach and decided to vaccinate after seeing others get the vaccine [36]. While this phenomenon has been analyzed by race and demonstrated among younger age groups, it has not (that we know of) been analyzed by older age or income [37]. Other variables that merit further exploration with regard to mortality include household crowding, which in early COVID-19 research was shown to be associated with infection rates in NYC. However, this research also demonstrated that neighborhood variables, including income levels, household crowding, and unemployment, were moderately to highly correlated [38].

## Conclusion

In the initial days of the vaccine rollout in New York, low COVID-19 vaccination coverage rates in low-income areas were frequently attributed by media, pundits, and even New York State’s governor to so-called vaccine hesitancy [36, 39, 40]. However, the fact that all wealth quintiles in NYC had a median coverage of over 87% once there was sufficient supply and community-based distribution suggests that vaccine access prevented higher coverage in low-income areas.

The study underscores the ethical implications of vaccine distribution strategies, highlighting the need for equity-focused approaches that include wealth and accessibility into risk-based formulas. Its findings can inform future strategies to prioritize high-risk groups, ensuring fair and effective public health interventions. Next time policymakers would do better to focus on the most vulnerable by deploying a “precision public health” approach.

## Data Availability

Data are available by request from the New York City Department of Health through the Open Data initiative.

## References

[1] FDA. *FDA takes key action in fight against COVID-19 by issuing emergency use authorization for first COVID-19 vaccine*. FDA; 2020. Accessed July 19, 2023. https://www.fda.gov/news-events/press-announcements/fda-takes-key-action-fight-against-covid-19-issuing-emergency-use-authorization-first-covid-19

[2] New York State. *Governor Cuomo Announces New Yorkers 30 years of age and older will be eligible to receive COVID-19 vaccine.* New York State; 2021. Accessed July 17, 2023. https://www.governor.ny.gov/news/governor-cuomo-announces-new-yorkers-30-years-age-and-older-will-be-eligible-receive-covid-19#:~:text=%22Today%20we%20take%20a%20monumental,House%2C%22%20Governor%20Cuomo%20said

[3] New York State. *Governor Cuomo announces state vaccination sites now open.* New York State; 2021. Accessed July 17, 2023. https://www.governor.ny.gov/news/governor-cuomo-announces-state-vaccination-sites-now-open

[4] Chow D, Mhaidli S. *Supply shortages, registration issues: NYC struggles with vaccine distribution*. NBC News; 2021. Accessed February 20, 2024. https://www.nbcnews.com/science/science-news/supply-shortages-registration-issues-nyc-struggles-vaccine-distribution-n1254446

[5] Roubein R, Ehley B. *States’ new vaccine worry: not enough doses*. Politico; 2021. Accessed February 20, 2024. https://www.politico.com/news/2021/01/20/states-coronavirus-vaccine-shortages-460899

[6] Fung K. *The digital divide is keeping many NYC seniors from scheduling COVID-19 Vaccinations.* Gothamist; 2021. Accessed July 17, 2023. https://gothamist.com/news/digital-divide-keeping-many-nyc-seniors-scheduling-covid-19-vaccinations

[7] Kim J. *The Vaccine Hurdles Older New Yorkers Face.* The New York Times; 2021. Accessed July 17, 2023. https://www.nytimes.com/2021/01/15/nyregion/covid-vaccine-nyc.html

[8] Mogul F. *New York City has been slow to vaccinate homebound elderly, causing more sickness.* NPR; 2021. Accessed July 17, 2023. https://www.npr.org/sections/health-shots/2021/06/16/1007138052/new-york-city-has-been-slow-to-vaccinate-homebound-elderly-causing-more-sickness

[9] Otterman S. *The maddening red tape facing older people who want the vaccine.* The New York Times; 2021. Accessed July 17, 2023. https://www.nytimes.com/2021/01/14/nyregion/covid-vaccine-older-people-senior-citizens.html

[10] New York State. *New York state statewide COVID-19 fatalities by age group.* New York State; 2023. Accessed July 17, 2023. https://health.data.ny.gov/Health/New-York-State-Statewide-COVID-19-Fatalities-by-Ag/du97-svf7/data

[11] Greer S, Adams L, Toprani A, et al. *The health of older adults in New York City.* NYC Health; 2019. Accessed July 18, 2023. https://www.nyc.gov/assets/doh/downloads/pdf/episrv/2019-older-adult-health.pdf

[12] Tejada-Vera B, Kramarow EA. *COVID-19 mortality in adults aged 65 and over: United States, 2020.* Centres for Disease Control and Prevention; 2022. Accessed July 17, 2023. https://www.cdc.gov/nchs/data/databriefs/db446.pdf 36256450

[13] McGowan VJ, Bambra C. COVID-19 mortality and deprivation: pandemic, syndemic, and endemic health inequalities. *Lancet Public Health*. 2022;7(11):966–75. 10.1016/S2468-2667(22)00223-7. 10.1016/S2468-2667(22)00223-7 PMC9629845 36334610

[14] Sepulveda ER, Brooker AS. Income inequality and COVID-19 mortality: age-stratified analysis of 22 OECD countries. *SSM - Popul Health*. 2021;16:100904. 10.1016/j.ssmph.2021.100904. 34584934 10.1016/j.ssmph.2021.100904 PMC8456048

[15] Yanez ND, Weiss NS, Romand J, et al. COVID-19 mortality risk for older men and women. *BMC Public Health*. 2020;20:1742. 10.1186/s12889-020-09826-8. 33213391 10.1186/s12889-020-09826-8 PMC7675386

[16] von Elm E, Altman DG, Egger M, et al. The strengthening the reporting of observational studies in epidemiology (STROBE) statement: guidelines for reporting observational studies. *Ann Intern Med*. 2007;147(8):573–7. 10.7326/0003-4819-147-8-200710160-00010. 17938396 10.7326/0003-4819-147-8-200710160-00010

[17] NYC Health. *Covid-19 Data - Vaccines*. New York City; 2021. Accessed July 17, 2023. https://www.nyc.gov/site/doh/covid/covid-19-data-vaccines.page

[18] New York City. *NYC Open Data.* The city of New York; n.d. Accessed July 17, 2023. https://opendata.cityofnewyork.us/

[19] American Community Survey 5-Year Summary File - Minnesota geospatial commons. Accessed July 4, 2023. https://gisdata.mn.gov/dataset/us-mn-state-metc-society-census-acs

[20] NYC Health. *Nychealth/Coronavirus-Data*. NYC Health.; 2023. Accessed July 17, 2023. https://github.com/nychealth/coronavirus-data/blob/master/trends/deaths-by-day.csv

[21] United States Census Bureau. *American Community Survey 5-Year Data (2009-2021).* United States Census Bureau.; 2023. Accessed July 5, 2023. https://www.census.gov/data/developers/data-sets/acs-5year.html

[22] Donnelly F. *The trouble with zip codes: solutions for data analysis and mapping.* At These Coordinates; 2020. Accessed August 23, 2023. https://atcoordinates.info/2020/05/11/the-trouble-with-zip-codes-solutions-for-data-analysis-and-mapping/. Accessed August 23, 2023.

[23] Anselin L. *Spatial econometrics: methods and models.* Dordrecht: Kluwer Academic Publishers; 1988.

[24] NYU Furman Center. *Sheepshead Bay BK15*. NYU Furman Center; 2022. Accessed July 17, 2023. https://furmancenter.org/neighborhoods/view/sheepshead-bay

[25] Khullar D, Chokshi DA. *Health, income, & poverty: where we are & what could help*. Health Affairs; 2018. Accessed July 5, 2023. https://www.healthaffairs.org/do/10.1377/hpb20180817.901935/full/

[26] Schwalbe N. *The US now has vaccines but no strategy on how to use them to defeat coronavirus.* The BMJ Opinion; 2020. Accessed July 23, 2023. https://blogs.bmj.com/bmj/2020/12/17/the-us-now-has-vaccines-but-no-strategy-on-how-to-use-them-to-defeat-coronavirus/

[27] Centres for Disease Control and Prevention. *People with certain medical conditions.* Centres for Disease Control and Prevention; 2023. Accessed July 5, 2023. https://www.cdc.gov/coronavirus/2019-ncov/need-extra-precautions/people-with-medical-conditions.html

[28] Albani VVL, Loria J, Massad E, Zubelli JP. The impact of COVID-19 vaccination delay: a data-driven modeling analysis for Chicago and New York City. *Vaccine*. 2021;39(41):6088–94. 10.1016/j.vaccine.2021.08.098. 34507859 10.1016/j.vaccine.2021.08.098 PMC8405507

[29] Davies JB. Economic inequality and COVID-19 deaths and cases in the first wave: a cross-country analysis. *Can Public Policy*. 2021;47(4):537–53. 10.3138/cpp.2021-033. 36039094 10.3138/cpp.2021-033 PMC9395158

[30] Pfizer. *Pfizer and BioNTech announce vaccine candidate against COVID-19 achieved success in first interim analysis from phase 3 study.* Pfizer.; 2020. Accessed August 7, 2023. https://www.pfizer.com/news/press-release/press-release-detail/pfizer-and-biontech-announce-vaccine-candidate-against

[31] Zhong X, Zhou Z, Li G, Kwizera MH, Muennig P, Chen Q. Neighborhood disparities in COVID-19 outcomes in New York city over the first two waves of the outbreak. *Ann Epidemiol*. 2022;70:45–52. 10.1016/j.annepidem.2022.04.008. 35487451 10.1016/j.annepidem.2022.04.008 PMC9042413

[32] Khoury MJ, Iademarco MF, Riley WT. Precision public health for the era of precision medicine. *Am J Prev Med*. 2016;50(3):398–401. 10.1016/j.amepre.2015.08.031. 26547538 10.1016/j.amepre.2015.08.031 PMC4915347

[33] Jimenez J. *NYC care: closing the healthcare access gap for undocumented New Yorkers*. The Joint Commission; 2023. Accessed February 20, 2024. https://www.jointcommission.org/resources/news-and-multimedia/blogs/advancing-health-care-equity/2023/01/nyc-care-closing-the-healthcare-access-gap-for-undocumented-new-yorkers/

[34] Thompson WC. *Getting in the door: language barriers to health services at New York City’s hospitals*. City of New York; 2005. Accessed February 20, 2024. https://comptroller.nyc.gov/wp-content/uploads/documents/jan10-05_geting-in-the-door.pdf

[35] Office of Disease Prevention and Health Promotion. *Access to Health Services*. U.S. Department of Health Services; n.d. Accessed February 20, 2024. https://health.gov/healthypeople/priority-areas/social-determinants-health/literature-summaries/access-health-services

[36] Kumar D, Mathur M, Kumar N, Rana RK, Tiwary RC, Raghav PR, Kumar A, et al. Understanding the phases of vaccine hesitancy during the COVID-19 pandemic. *Isr J Health Policy Res*. 2022;11:16. 10.1186/s13584-022-00527-8. 35317859 10.1186/s13584-022-00527-8 PMC8939479

[37] Adams SH, Schaub JP, Nagata JM, Park MJ, Brindis CD, Irwin CE Jr. Young Adult Perspectives on COVID-19 Vaccinations. *J Adolesc Health*. 2021;69(3):511–4. 10.1016/j.jadohealth.2021.06.003. 34274212 10.1016/j.jadohealth.2021.06.003 PMC8277980

[38] Emeruwa UN, Ona S, Shaman JL, et al. Associations between built environment, neighborhood socioeconomic status, and SARS-CoV-2 infection among pregnant women in New York City. *JAMA*. 2020;324(4):390–2. 10.1001/jama.2020.11370. 32556085 10.1001/jama.2020.11370 PMC7303894

[39] CBS New York. *Gov. Cuomo urges New Yorkers to get COVID vaccine: ‘We’ll Make It Accessible, but We Need You to Accept It’.* CBS New York; 2021. Accessed July 5, 2023. https://www.cbsnews.com/newyork/news/governor-cuomo-nyc-covid-vaccine/

[40] Sanchez JM, Wilkinson O. *COVID-19 Death gap by county income widened after vaccine availability.* Federal Reserve Bank of St. Louis; 2022. Accessed July 23, 2023. https://www.stlouisfed.org/publications/regional-economist/2022/mar/covid19-death-gap-county-income-widened-vaccine-availability

